# Effect of Particle Size and Surface Charge on Nanoparticles Diffusion in the Brain White Matter

**DOI:** 10.1007/s11095-022-03222-0

**Published:** 2022-03-21

**Authors:** Tian Yuan, Ling Gao, Wenbo Zhan, Daniele Dini

**Affiliations:** 1 Department of Mechanical Engineering, Imperial College London, London, SW7 2AZ UK; 2 School of Biomedical Engineering and Imaging Sciences, King’s College London, St. Thomas Hospital, London, SE1 7EH UK; 3 School of Engineering, King’s College, University of Aberdeen, Aberdeen, AB24 3UE UK

**Keywords:** Brain diseases, Brain tissue, Diffusion coefficient, Extracellular space, Nanoparticles

## Abstract

**Purpose:**

Brain disorders have become a serious problem for healthcare worldwide. Nanoparticle-based drugs are one of the emerging therapies and have shown great promise to treat brain diseases. Modifications on particle size and surface charge are two efficient ways to increase the transport efficiency of nanoparticles through brain-blood barrier; however, partly due to the high complexity of brain microstructure and limited visibility of Nanoparticles (NPs), our understanding of how these two modifications can affect the transport of NPs in the brain is insufficient.

**Methods:**

In this study, a framework, which contains a stochastic geometric model of brain white matter (WM) and a mathematical particle tracing model, was developed to investigate the relationship between particle size/surface charge of the NPs and their effective diffusion coefficients (*D*) in WM.

**Results:**

The predictive capabilities of this method have been validated using published experimental tests. For negatively charged NPs, both particle size and surface charge are positively correlated with *D* before reaching a size threshold. When Zeta potential (Zp) is less negative than -10 mV, the difference between NPs’ *D* in WM and pure interstitial fluid (IF) is limited.

**Conclusion:**

A deeper understanding on the relationships between particle size/surface charge of NPs and their *D* in WM has been obtained. The results from this study and the developed modelling framework provide important tools for the development of nano-drugs and nano-carriers to cure brain diseases.

## Introduction

Nanoparticles (NPs), which are characterised by a diameter in the range of a few nanometres, have become a promising drug delivery system for the treatments against various brain disorders, owing to the ability to cross the blood-brain barrier (BBB) [1]. A variety of materials have been applied to fabricate NPs, ranging from natural and synthetic polymers, metals to lipid-based or carbon-based materials. Such a wide selection enables the NPs to be tailored with desired chemical and physical characteristics to fulfil the specific delivery purposes [2]; these include BBB penetration, controlled release, sustainable drug supply and localised delivery [3–5], etc.

NP transport in brain tissues is dominated by diffusion [6]. Effective diffusion coefficient (*D*) is a measure of the rate at which the NPs can spread in the tissue. A high value of *D* usually indicates a short time window for transport. Several efforts have been made to increase the *D* of NPs, such as modifying the particle size to obtain a higher ratio of molecular thermal motion to the resistance [7], and charging the NP surface to avoid aggregation and deposition [8, 9]. These means have been adopted to enhance the BBB penetration of NPs [10–12]. However, whether these modified NPs with the enhanced BBB penetration also have higher effective *D*s in the brain parenchyma cannot be guaranteed, because the anatomical structures of BBB and brain parenchyma are very different.

Some studies have provided insights on the important roles that particle size and surface charge can play on NPs diffusion in the brain parenchyma. For example, by measuring *D*s of uncharged NPs in rat brain neocortical regions, Thorne *et al.* [13] concluded that the width of brain tissue extracellular space (ECS) is about 38~64 nm. And the experimental results also showed the negative correlation between particle size and *D* of the NPs. However, these results are only applicable for uncharged NPs. Years later, Nance *et al.* [14] found that NPs as large as 114 nm in diameter were also able to transport inside rat and human brain if they were coated with dense poly(ethylene glycol) (PEG), which charged the NPs by about -5 mV. Moreover, Nance and co-workers also demonstrated that different surface functionalities of polystyrene (PS) [14], poly(lactic-co-glycolic acid) (PLGA) [15], dendrimer [16], and quantum dot [17], which charge these NPs with different Zp and also change their hydraulic diameters, led to different diffusion behaviours of the NPs within the brain parenchyma. In the experiment of Dal *et al.* [18], where apolipoprotein E4 was adsorbed onto polysorbate 80-stabilized NPs and charged the surface by -10 mV, the brain accumulation of the NPs was also improved by 3 folds compared with unmodified NPs. These experimental investigations highlighted the difference made by surface modification of NPs on their brain diffusion.

Nevertheless, by analysing the experimental data reported in the literature, it is evident that there is a gap in the knowledge about the mutual influence and the possibility to decouple the effect of these two parameters (particle size and surface charge) in order to understand their independent effect. Although we now have known that smaller and negatively charged NPs normally possess higher *D* than bigger and electroneutral NPs, no study has confirmed if there exist exact thresholds for the two parameters. In addition, it is also not clear if one of these two parameters obliterates the other. For example, it may be less intuitive to judge whether the *D* will increase or decrease when an end functional group gives a NP a bigger size but more negative Zp. Filling this gap of understanding is important to promote the design efficiency of NPs, but it is not easy to perform by experiments only, because particle size and surface charge always change simultaneously after surface functionalization. Take PEG and COOH, two commonly used end functional groups for NPs, as an example; while PEG nearly does not charge NPs and COOH charges NPs negatively, PEG-coated NPs are generally 10 to 20 nm larger than the COOH-coated NPs [14]. Structural complexity and limited accessibility of brain tissue, difficulties in precise control of NPs’ parameters, and low visibility of NPs [18] also make it less feasible to conduct quantitative studies by experiments. By contrast, mathematical modelling is a good alternative to easily decouple these two parameters and provide insights into the abovementioned concerns.

White matter (WM) acts as a relay station and transmits messages between different parts within the central nervous system [19]. As a result, diseases with white matter, such as Alzheimer's disease and glioblastoma, can critically affect brain function [20]. However, transport of NPs, which is a promising technique to treat these diseases, in WM has not received sufficient attention. In addition, owing to the ordered distribution of axons that compose WM and the development of new analytical techniques, computational resources and image analyses methodologies, geometrical reconstruction of the WM’s detailed microstructure becomes feasible by programming [21, 22]. Therefore, in this paper, a microstructural model of WM is reconstructed to mimic the microenvironment of brain tissue, where the NPs transport occurs. A mathematical model is also built to trace the trajectory of every single particle in this realistic virtual prototype of WM, the result of which can be used to calculate the *D* of NPs [23]. Based on this framework, both independent and coupling effects of NP’s size and Zp on its *D* are investigated, which can be used to improve our ability to design NPs for the treatment of brain diseases.

## Materials and Methods

Eq. (1) maps the macroscopic quantity (diffusion coefficient) to the microscopic mechanism (displacement of particles) and provides a generalised method to calculate the diffusion coefficient of particles [23, 24], which requires the trajectory of every single particle to be obtained as the input.

1 $${\displaystyle \begin{array}{c}{D}_0=<{R}^2>/6t\\ {}<{R}^2>=\sum\limits_{i=1}^n\left(d{x}_i^2+d{y}_i^2+d{z}_i^2\right)\end{array}}$$

Where *D*_0_ is the diffusion coefficient of particles governed by Brownian motion. <*R*^2^> is the average of mean square displacement (MSD) of all the particles. *dx*, *dy*, *dz* are the displacement of a NP in *x*, *y*, *and z* direction, respectively. *n* is the number of NPs in the system. *t* is the diffusion time.

Therefore, in this section, a mathematical model is built to trace the NPs in the ECS of WM. To take account the specificity of brain’s microstructure, a stochastic microstructural models of brain WM is built and used as the geometry.

### Mathematical model

If treated as an individual entity, the forces acting on a NP that affect its trajectory are from (i) its own molecular thermal motion, (ii) interaction with the ambient fluid environment, (iii) interaction with the surrounding particles, and (iv) interaction with the cell membrane [25]. Thus, to track the trajectory of every single NP, these 4 types of forces need to be specified first.

#### Molecular thermal motion - Brownian motion

By modelling the Brownian motion as a Gaussian white noise process, the mathematical expression of Brownian force can be written as Eq. (2) [26]:

2 $${\mathbf{F}}_{\mathrm{B}}=\Phi \sqrt{\frac{12\pi {K}_{\mathrm{B}}\mu T{r}_{\mathrm{p}}}{\Delta t}}$$

where **F**_B_ is the Brownian force, *k*_B_ is the Boltzmann constant, μ is the dynamic viscosity of the fluid, *T* is the absolute temperature of the fluid, ∆*t* is the time step, Φ is a Gaussian random number with zero mean and unit variance to take the randomness of Brownian motion into account, *r*_p_ is the radius of the particle.

#### Forces from the fluid environment

The viscous drag force on the particles due to fluid resistance is modelled using Stokes' Law [27], as given by:

3 $${\mathbf{F}}_{\mathrm{D}}=6\pi \mu {r}_{\mathrm{p}}\left({\mathbf{v}}_{\mathrm{flow}}-{\mathbf{v}}_{\mathrm{p}\mathrm{article}}\right)$$

where **F**_D_ is drag force, **v**_flow_ is the velocity of fluid flow, and **v**_particle_ is the velocity of the particle.

In the applications related to NPs, certain adaptations are needed to modify Eq. (3). The coefficient defined by Eq. (4) considers the slip boundary effects at the particle-fluid interface in terms of nano-sized particles:

4 $${C}_{\mathrm{slip}}=1+2{K}_{\mathrm{n}}\left(1.257+0.4{e}^{-\frac{1.1}{2{K}_{\mathrm{n}}}}\right)$$

where *K*_n_ is the Knudsen number and is set as 0.025 in this work [28]. This correlation is valid up to *Re* = 800. *C*_slip_ becomes important when the size of the particles is less than 15 nm [28].

Substituting Eqs. (4) into Eq. (3), the equation of drag force for NPs can be rewritten as:

5 $${\mathbf{F}}_{\mathrm{D}}=6\pi \mu {r}_{\mathrm{p}}\left({\mathbf{v}}_{\mathrm{flow}}-{\mathbf{v}}_{\mathrm{p}\mathrm{article}}\right)/{C}_{\mathrm{slip}}$$

Due to the small size of NPs, the buoyancy force and gravity of the NPs are small enough to be neglected [25].

#### Forces from the surrounding particles

NP aggregation not only reduces the particle’s diffusivity but also causes particle deposition. To avoid such phenomena, the NP surface is usually charged to enable the particles repelling each other when getting close. Moreover, the NP solution is diluted in this study (see section 2.5). Therefore, the hard collision between particles could be neglected [29], and the electrostatic force dominates the particle-particle interaction. We therefore introduce Coulomb force, as shown in Eq. (6), to account for the forces from the surrounding particles.

6 $${\mathbf{F}}_{\mathrm{C}}=\frac{q{q}_i}{4\pi {\epsilon}_0}\sum_{i=1}^n\frac{r-{r}_i}{{\left|r-{r}_i\right|}^3}$$

where **F**_C_ is the Coulomb force, *q* is the charge quantity of the target particle, *q*_*i*_ is the charge quantity of one of the surrounding particles, *ϵ*_0_ is the vacuum permittivity, *r* and *r*_*i*_ stand for the position of the two particles.

Eq. (6) requires the charge quantity of particles needs to be known before calculating the **F**_C_, but unfortunately, the charge quantity of the NPs cannot be measured directly. The only measurable property related to the charge status of a particle is its Zeta potential (Zp), which is commonly used in NP characterisation [10, 30].

**Fig. 1:**
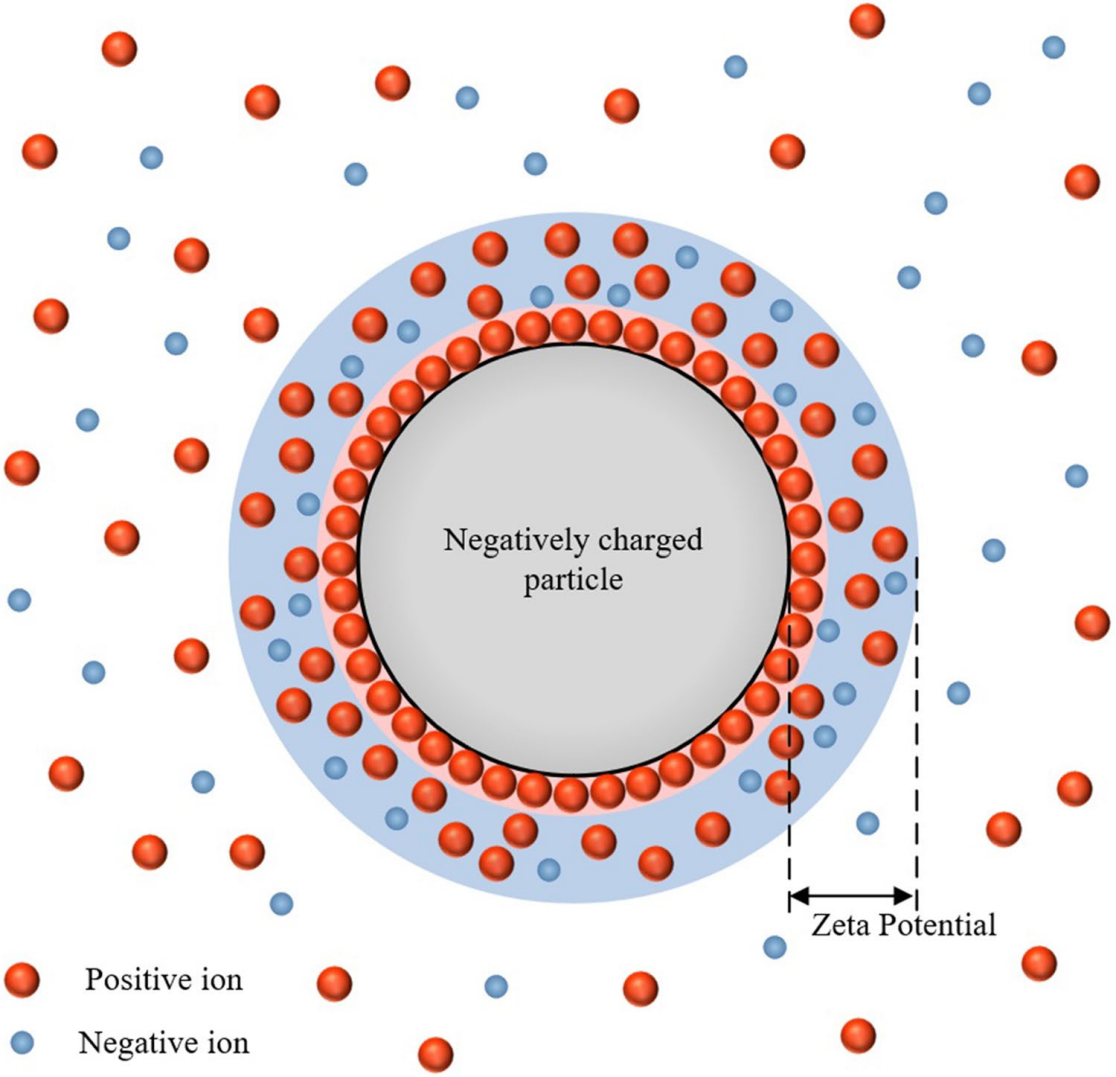
Relationship between surface charge density and Zp of a negatively charged particle.

There is a specific relationship between the surface charge density and Zp of NPs, as shown in Fig. 1. For instance, positive ions would strongly bind on the surface of negative charged NPs to form a stern layer (pink). Outside this layer, both positive and negative ions are loosely attached to form the diffusion layer (blue). The effects of NP on ions would decrease to the neglectable level outside the diffusion layer, where ions could move freely. Zp is defined as the electric potential between the particle surface and the outside surface of the diffusion layer. This form the core theory of electric double layer (EDL) [31].

By solving the 1D Possion-Boltzmann equation, the Gouy-Chapman equation (Eq. (7)) could be obtained to quantify the relationship between surface charge density (*δ*) and surface electrostatic potential (*Ψ*_0_) of a flat charged surface [32]. The same relationship exists between the effective charge density (*δ*_eff_) and Zp (*ζ*) of a particle, as shown in Eq. (7). However, being simplified to be flat surface, the effective charge density (*δ*_eff_) of a sphere particle is not equal to its real surface charge density (*δ*). With the aid of molecular dynamics simulations, Ge *et al.* [33] successfully estimated the nano diamond surface charge density (*δ*) from its measured Zp and drew the relationship between the effective surface charge density (*δ*_eff_) and real surface charge density (*δ*) for nano-diamond to be some curves, as shown in Fig. 4(a) in Ref. [33]. Given the shape of nano-diamond is closer to sphere than the shape of plates, this relationship is adopted in this study for a more precise description of the effective charge density (*δ*_eff_) of NPs.

7 $$\delta =\sqrt{8 cN\epsilon {k}_{\mathrm{B}}T}\sinh \frac{e{\Psi}_0}{2{k}_{\mathrm{B}}T}\kern5em \mathrm{for}\ \mathrm{flat}\ \mathrm{plate}$$

8 $${\delta}_{\mathrm{eff}}=\sqrt{8 cN\epsilon {k}_{\mathrm{B}}T}\sinh \frac{e\upzeta}{2{k}_{\mathrm{B}}T}\kern4.75em \mathrm{for}\ \mathrm{NP}$$

where *c* stands for the ion concentration, *ϵ* is the permittivity of the solution, *N* is the Avogadro constant, *δ* and *Ψ*_0_ are the surface charge density and surface electrostatic potential of a flat charged surface, respectively.

#### Forces from the cell membrane

As the cell membranes are usually negatively charged because of the lipid bilayer [34], particle-surface interactions become significant when the charged NPs move into the active range of cell membranes. To define the force acting on the particle from the surface, Derjaguin, Landau, Verwey, and Overbeek (DLVO) theory [35] was adopted:

9 $${\mathbf{F}}_{\mathrm{potential}}=-\frac{\partial }{\partial h}\left({A}_{\mathrm{elec}}+{A}_{\mathrm{vdw}}\right)$$

where *A*_elec_ is the potential induced by electrostatic interaction and *A*_vdw_ =  − *A*_H_*d*_p_/(12*h*) is the potential induced by van der Waals force. *A*_H_ is the Hamaker constant that can be calculated by an empirical formulation provided by the reference [36], *d*_p_ is the diameter of the NP, and *h* is the distance between the NP and the cell membrane.

Given the size of NPs is several orders lower as compared to the axons, the cell membrane can be treated as a flat plate [25]. Thus, the Gouy–Chapman equation (Eq. (7)) can be used to calculate the electric potential, *i.e.*, *A*_elec_ = Ψ_0_.

#### Other factors and inherent model approximations

It should be noted that the abovementioned 4 forces cannot fully represent the brain microenvironment, with other complex local hydraulic and electrostatic factors which should also be considered to complete an accurate picture. These include local fluid flow due to water transport across the membranes, local charge gradients, extracellular matrix components which add steric and electrostatic effects, hydrogen bonding, hydrophobic interactions, local osmotic gradients due to the ion exchange in extracellular matrix, and the local viscosity change due to these components as well as steric and adhesive interactions. A comprehensive review on the key mechanisms governing molecular interactions and NPs flow in brain can be found in Ref. [37].

However, impacts of these factors on NPs’ trajectory are too complex to be simultaneously described an d solved mathematically within the current framework and the scope of this contribution. Even if some factors might be added in the g overning equations, such as the local viscosity, further experimental characterisation of the corresponding viscosity gradient distribution is needed. Yet these experimental data are not available. In addition, since our focus is the effect of particle size and Zp on NP diffusion, the factors that cannot be mathematically described could be treated as a lumped system [38], which means that although every one of the factors inside the lumped system may manipulate the movement of the particles in a specific manner, the overall effect of this system on every single particle is the same. Under this condition, this overall effect could be represented, in first approximation, by a single parameter, such as the global viscosity.

#### Particle trajectory

With all the major forces obtained from the above, the trajectory of every single particle can then be calculated by Newton’s second law:

10 $$d{\mathbf{r}}_{\mathbf{i}}=\left[\sum\limits_{i=1}^N\left({\mathbf{F}}_{\mathrm{B}i}+{\mathbf{F}}_{\mathrm{D}i}+{\mathbf{F}}_{\mathrm{C}i}+{\mathbf{F}}_{\mathrm{potential}i}\right)\right]{\left(\Delta t\right)}^2$$

where *d***r**_**i**_ is the displacement vector of the *i*th particle, ∆*t* is the time interval used in the integration.

### Geometric model

Brain is mainly composed of neuronal cells, with the cell bodies forming grey matter (GM) and the nervous fibres (axons) constituting WM. In WM, the axons are randomly distributed in the transverse direction but have a uniform orientation along the longitudinal direction [39]. Although the size of every single axon is not determinate, the diameters of all the axons follow a prescribed probability distribution [40, 41]. Based on these rules, a two-dimensional geometric model is built in this study using an in-house MATLAB program based on the statistical geometrical data of WM (see Table I) to mimic the cross section of WM.

**Table I:** Parameters used in this study

Parameter	Value	Unit	References
Tissue porosity	0.3	-	[21]
Axonal distance (mean)	0.1	μm	[13, 21]
Axonal length (mean)	15	μm	[53]
Axonal diameter (mean)	1	μm	[41]
Cell surface Zp	-20	mV	[54]
Temperature (T)	310	K	[13]
Viscosity (Dilute agarose)	6.9152×10^-4^	Pa∙s	[13]
Viscosity (IF)	3.5×10^-3^	Pa∙s	[55]
Elementary charge (*e*)	1.60×10^-19^	C	[56]
Permittivity of NS (*ϵ*)	6.55×10^-10^	C/Vm	[57]
Vacuum permittivity (*ϵ*_0_)	8.85×10^-12^	C/Vm	[58]
Avogadro constant (*N*)	6.02×10^-23^	mol^-1^	[59]
Boltzmann’s constant (*k*_B_)	1.38×10^-23^	J/K	[60]

**Fig. 2:**
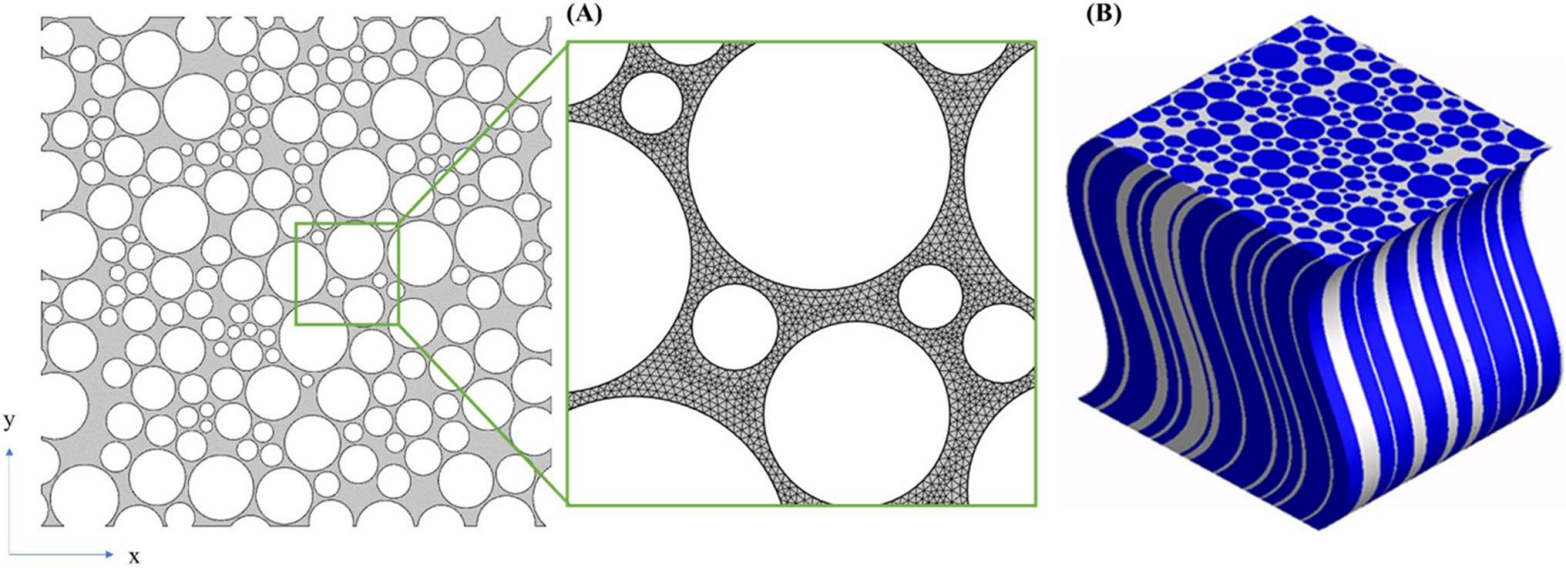
**(A)** Stochastic geometric model used in this study and the mesh density for simulations. The void circles represent the axons, and the grey domain stands for the ECS. **(B)** Simplified 3D geometry of WM used in mechanical or hydraulic simulations. The blue elements represent axons and the white space is ECS.

As shown in Fig. 2(A), the axons are simplified as randomly distributed circles, with the diameters randomly selected from the experimental range [41]. The program [21] ensures that (i) the average diameter of all the axons is 1 μm [41]; (ii) the minimum distance between every two axons is 0.1 μm [13, 21]; (iii) no overlap exists between any two axons, and (iv) the overall porosity of the domain is 0.3 [21]. These are to reproduce the four essential geometrical properties that govern the transport properties in the ECS, namely axon diameter distribution, ECS volume ratio, distance between axons, and spatial organisation of the tissue. In fact, the representative parameters used for geometrical reconstruction would vary considerably depending on the location in brain, as the microstructure varies significantly across the brain; and the entire body of literature generated by the diffusion-weighted MRI field has highlighted challenges for drug delivery in the brain due to regional variation [42]. Age, gender, and health condition also make the microstructures of brain to be different [43].

The dimension of this model is 18×18 μm which has been demonstrated to be large enough to contain sufficient axons to form a representative volume element (RVE) for brain WM [21]. The non-structural element is used, and the mesh size is determined by conducting a mesh sensitivity study through varying the number of elements used to model the space between axons. It is found that at least 2 elements should be generated in the gap between any two axons, as shown in Fig. 2(A).

It should be noted that anisotropy is one of the major unique characteristics of WM compared with other regions of the brain due to the directional distribution of axons inside WM, and diffusion along the axons is faster than across the axonal direction [44]. Therefore, performing 3D simulation is very important. However, only the diffusion direction perpendicular to the axons is considered in this model because the longitudinal reconstruction of WM’s microstructure (geometry) is particularly challenging, and an accurate 3D reconstruction has escaped researchers so far; this is because the longitudinal shapes (curvatures) of axons are quite different while the distance between the axons is extremely close (1/10~1/20 of axon’s diameter) [13, 21]. Packing these 3D structures with mixed shapes in such a dense pattern without overlapping is technically very challenging. Some works elongate the cross-section to generate 3D geometry [22, 45], which means that all the axons share the same longitudinal shape and tortuosity, as shown in Fig. 2(B). This simplification provides a practical solution to this challenging problem and may be valid when modelling of mechanical properties; however, this is unjustified for the solution of the diffusion problem since longitudinal tortuosity of the axons has great impact on the particle’s diffusivity [46–48]. Some other more sophisticated geometric models also surfer from the similar concerns [49]. Regarding the concern that diffusion along the axons may dominate the diffusion tensor, in our previous work [44], which simulated particle diffusion in a straight elongated 3D geometry and Monte Carlo method, the results showed that diffusion along the axons (*D*_∥_) is about 1.3~1.9 times faster than across the axons (*D*_⊥_). Note that that work did not consider the longitudinal tortuosity of the axons, so the real ratio (*D*_∥_/ *D*_⊥_) should be even smaller. This implies that although diffusion in the parallel direction is faster than that in the perpendicular direction, *Ds* in both directions are important components of the *D* tensor. In addition, although the microstructures of the extracellular space in the two directions are not identical, the diffusion mechanisms and the types of force acting on the NPs are the same no matter in which direction they are diffusing. Because of the longitudinal torsion and bending of the axons, the NPs would also need to pass the similar narrow gaps when they diffusion along the axons. This means that the microstructure in both directions hinder the diffusion of NPs in the same way but modulates it by different magnitudes. As the aim of this work is to understand how particle size and surface potential of the NP affect it effective diffusion coefficient but not the accurate prediction of the diffusivity tensor, it is believed that using perpendicular and parallel geometric model will reach similar results in terms of their quantitative analysis. Considering all the above-mentioned concerns, we did not deal with the longitudinal direction in the present work. In our future works, we aim to tackle the issue of the reconstruction of an accurate 3D geometry for brain WM, and the mathematical model built in this paper could also be directly adopted to study 3D volumes.

### Material properties

The aim of this study is to calculate the self-diffusion coefficient of NPs, so the convection is not considered, and the fluid is assumed as static. To take into account EDL effects, the solution is set as normal saline (NS) with the ion concentration (*c*) of 0.154 mol/L [50]. Based on the practical applications in literature [51, 52], the NP size and Zp are located in the ranges of 20~98 nm and -50~0 mV, respectively. The other material and geometric parameters that are used in this study are summarised in Table I.

### Boundary conditions

Focusing on the measurement of diffusivity, endocytosis and release dynamics of NPs are not considered in the simulations. Therefore, it is assumed that the particle undergoes diffuse scattering once it contacts with the cell membrane. NPs would move out of the computational domain once they reach the boundaries. The electric potential of -20 mV [54] was assigned to all the cell membranes.

### Simulation setup

At *t* = 0 *s*, 900 NPs were released from a 0.2×0.2 μm^2^ square area at the centre of the model to mimic the transportation process of drug molecules from the injection site. This number of NPs is selected after a sensitivity study, which is adequate to obtain statistically stable results for calculating the MSD defined in Eq. (1). The corresponding initial concentration of the NPs was 0.37 mM which is within the NPs’ concentration of the solutions used for brain diffusion experiments [13].

COMSOL Multiphysics 5.6 software package [61] is used to solve the equations and calculate the trajectories of the NPs. The linear solver is set as Multifrontal Massively Parallel Sparse direct solver (MUMPS) and the Automatic Newton method is chosen as the nonlinear solver [62].

## Results

### Model verification

The present model is firstly compared to the experimental results reported in Ref. [13, 63]. In Ref. [13], *D*s of the water-soluble quantum dots with multiple diameters were measured in dilute (0.3%) agarose and rat neocortex (GM) *in vivo*. In Ref. [63], the *D*s of gadobutrol (cerebrospinal fluid (CSF) tracer with the hydraulic diameter of 2 nm) in both GM and WM were calculated based on MRI analysis and partial differential constrained optimization. Because of the structural difference between WM and GM, the experimental results in GM are not used for comparison.

**Fig. 3:**
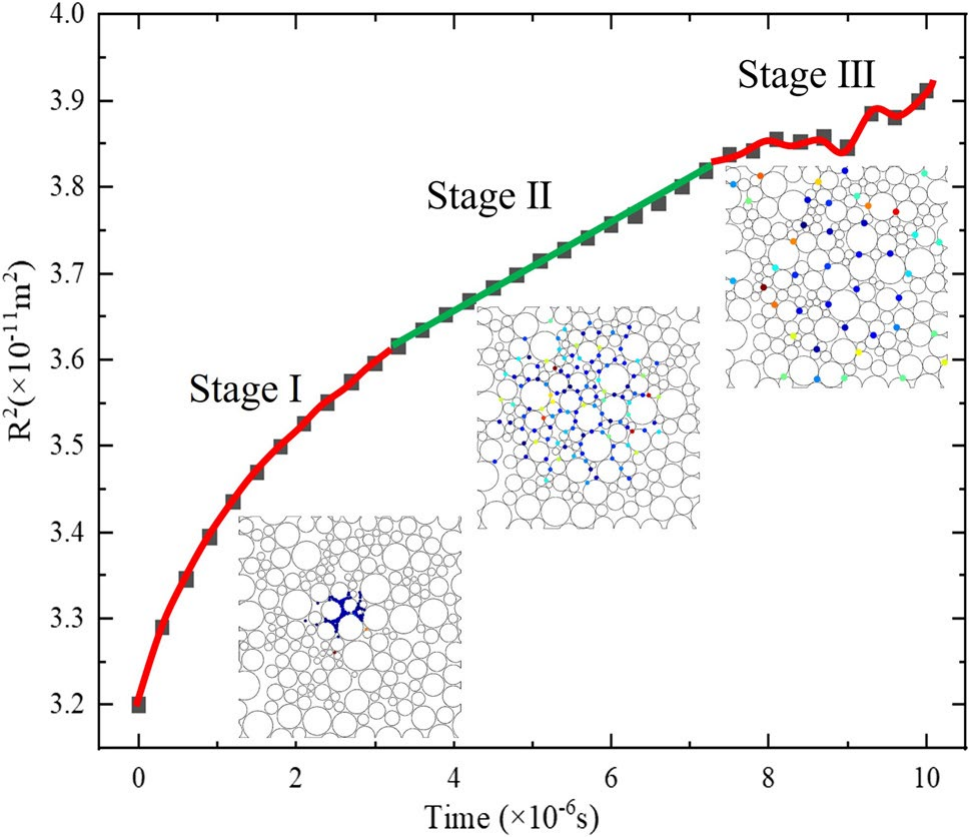
Typical *time* − *R*^2^ relationship of the NPs.

According to the simulation results, the typical diffusion process of the NPs can be divided into 3 stages, as shown in Fig.3. On stage I, as all the particles are released simultaneously from the centre of the model, the high particle concentration would result in significant interactions between particles and hence the high particle velocity. Therefore, the initial slope of the *time* − *R*^2^ curve is high. With time proceeding, the NPs disperse into the MW gradually. This consequently weakens the interactions among particles until reaching a statistically stable stage, where the *time* − *R*^2^ curve presents a constant slope, defined as Stage II. NPs would move farther and leave the computational domain. The lack of NP numbers in the domain would strongly reduce the stability of the statistical results and thereby cause the fluctuation of the *time* − *R*^2^ curve on Stage III, as shown in Fig. 3. Therefore, the slope of *time* − *R*^2^ curve on stage II is chosen to calculate the *D*. A similar trend was also observed in a recent experiment in which the diffusion of single carbon nanotube was tracked in rat organotypic hippocampal slices and acute brain slices from adult mice [64].

**Fig. 4:**
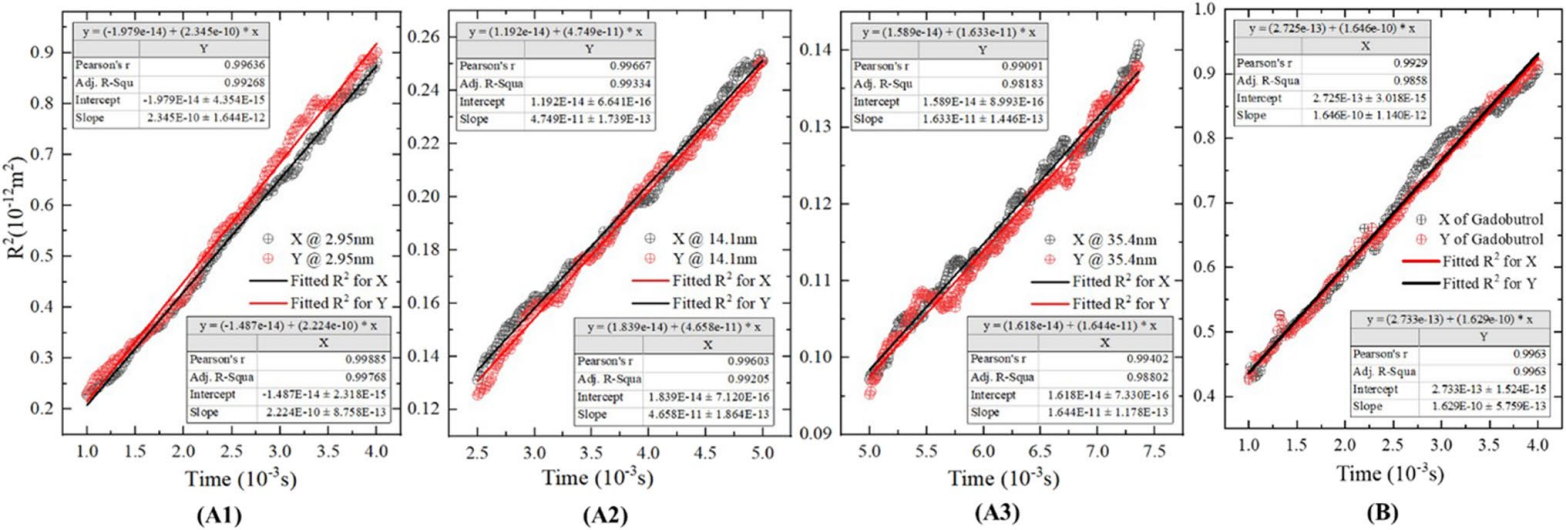
Method of calculating *D* and simulated results of experiments in Ref. [13, 63]. **(A)** Diffusion in dilute (0.3%) agarose. **(B)** Diffusion in WM.

The *Time* − *R*^2^ curve on Stage II is plotted based on the statistical results of all NPs’ trajectories in both the *x* and *y* direction (see Fig. 2 (A) for the directions) using Eq. (11). Linear curve fitting is used to calculate the curve slope, as shown in Fig. 4, which is then submitted into Eq. (12) [65].

11 $$<{R}_x^2>=\sum_{i=1}^nd{x}_i^2$$

12 $$D=<{R}_x^2>/2t$$

The results in Fig. 4 indicate that the model is nearly isotropic in the *x* and *y* directions as the slopes are nearly the same, confirming the tissue behaves as a transversely isotropic material [66]. Thus, the values of *D* in the *x* and *y* directions are averaged to represent the *D* in the direction perpendicular to the axons.

The calculated *D*s are then compared with the tested values in Table II. It shows that simulation results agree well with the tested results for the diffusion in agarose. The calculated *D*s are underestimated when comparing with that in WM. The main reason may come from (i) the calculated *D* is the perpendicular component of the diffusivity tensor whereas the tested *D* provides an estimate of the value of the whole tensor. Since parallel diffusivity (*D*_∥_) is about 1.3~1.9 times faster than perpendicular diffusivity (*D*_⊥_) [44], the value of whole tensor must be larger than the perpendicular component, and (ii) the factors that cannot be mathematically described as stated in section 2.1.5. The components in extracellular matrix (ECM), such as fibres and proteins, may prevent the movement of NPs by collisions in real brain; however, the local fluid flow due to water transport across the membranes may, on the other hand, accelerate the NPs. In fact, to reduce the effect of these simplifications as far as possible, the measured realistic viscosity of IF, as given in Table I, is used in the simulations of brain diffusion. This is to treat these complex factors as an effective change in viscous resistance on the particles.

**Table II:** Comparison between experimental measurements and simulation results

*d*_H_ (*nm*)	*D*, 10^−11^m^2^/s (Experiment)	*D*, 10^−11^m^2^/s (Simulation)
2.95	22.2±0.16 (Agarose)	22.8±0.13 (Agarose)
14.1	4.67±0.061 (Agarose)	4.70±0.018 (Agarose)
35.4	1.86±0.049 (Agarose)	1.64±0.013 (Agarose)
2.00	20±0.1 (WM)	16.39±0.01 (WM)

Furthermore, the trends of simulated results and test results are the same, which confirms that *D* decreases with the increase of particle size.

### Effect of Zp

**Fig. 5:**
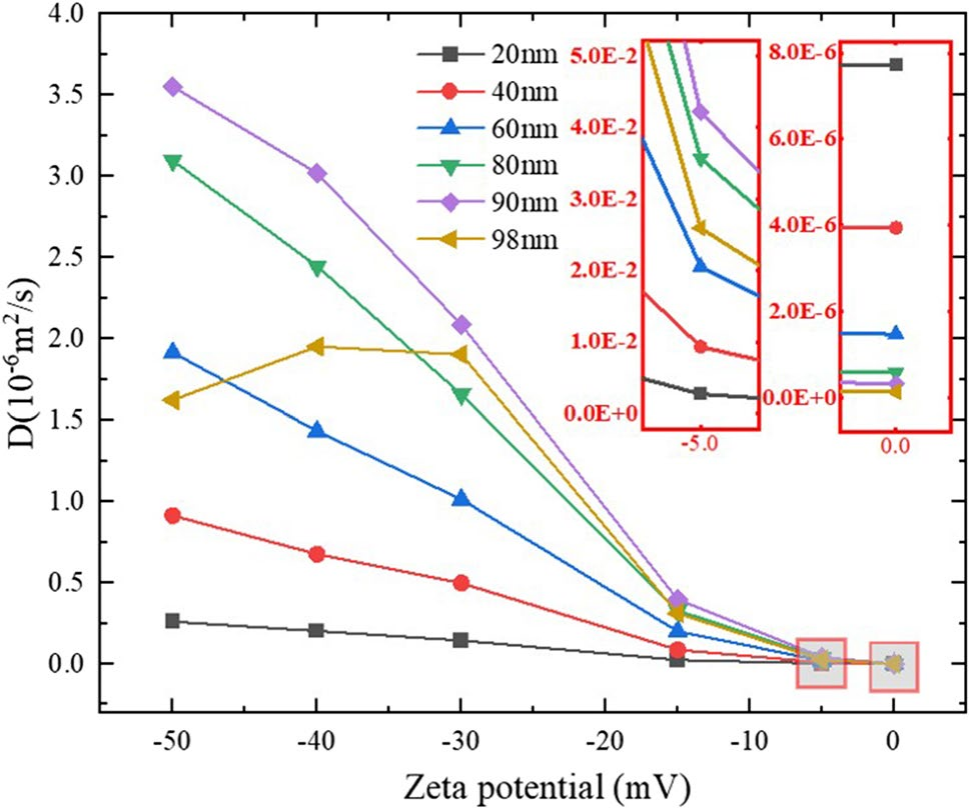
Relationship between Zp and *D*.

The Zp-*D* relationships are plotted in Fig. 5. Note that Zp is negative in this study. The results imply that increasing the absolute value of Zp can significantly increase *D*. For example, when Zp is 0 mV, the order of magnitude of *D* is 10^−13^ ∼ 10^−12^ m^2^/s. If the absolute value of Zp is increased by 5 mV, the order of magnitude of *D* is 10^−9^ ∼ 10^−8^ m^2^/s, which is 10,000 times larger than that of the uncharged NPs. If Zp is more negative, *D* can be further increased but stay in the same order of magnitude.

An interesting phenomenon is the different behaviour observed for the 98 *nm* particles. While the *D* of other particles is increasing continually with the increase of the absolute value of Zp, the *D* of 98 nm particles begin to decrease when the Zp is more negative than 30 mV. With a more negative Zp, a NP will have a higher surface charge, and consequently, a higher kinetic energy due to the larger Coulomb force between particles. Therefore, it is reasonable that *D* increases with the absolute value (negative) of Zp. However, when the particle size is close to the size of the channel (the distance between two axons, which is 100 nm in average), there is little space for the particles to pass through. Under this circumstance, higher kinetic energy means higher chance of particle-cell collisions and lower possibility for the particle to pass through the narrowest gaps. Therefore, when the particle size is very close to the average width of the channel, a high absolute value (negative) of Zp may even decrease the *D* of NPs.

### Effect of particle size

**Fig. 6:**
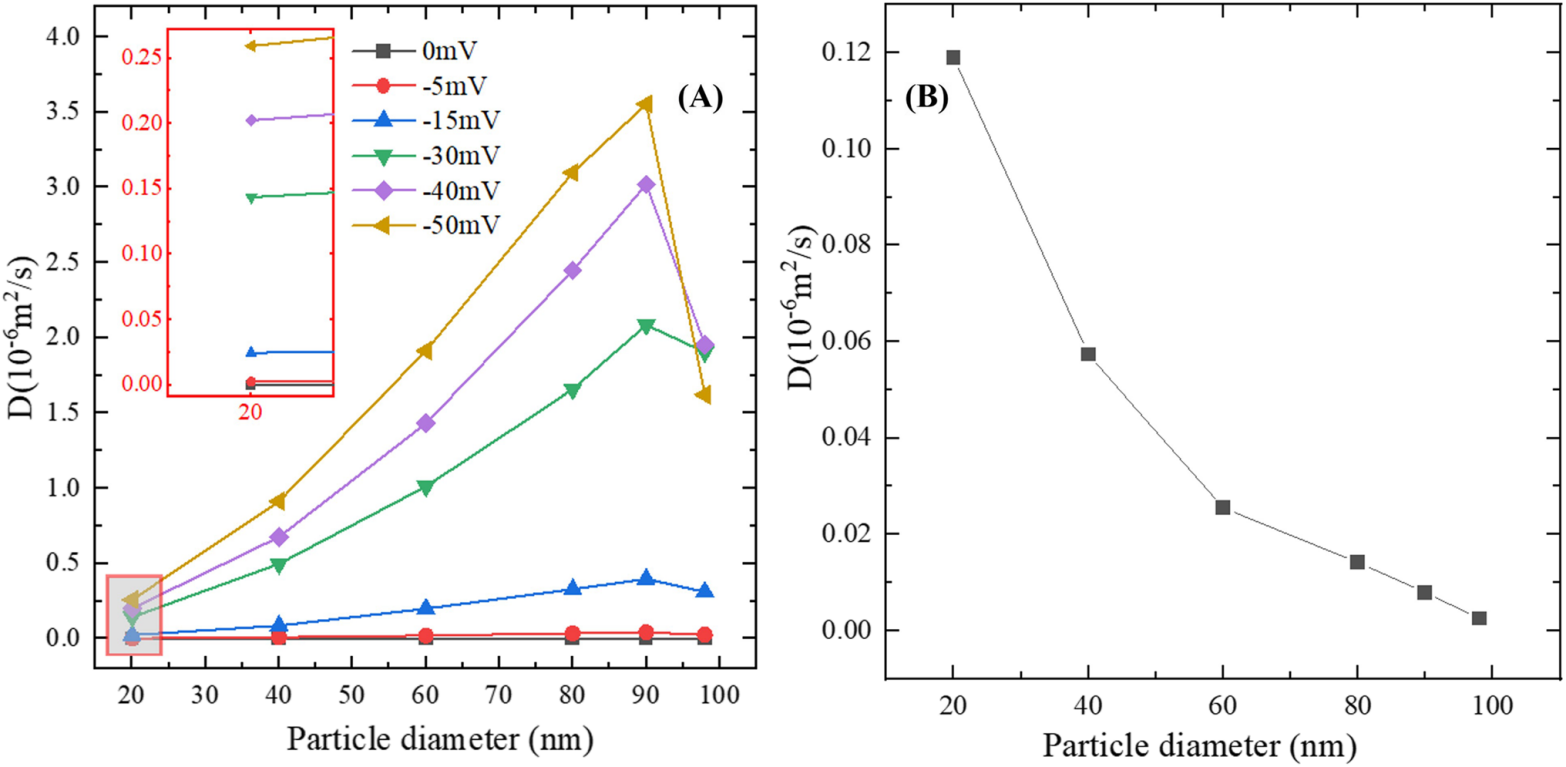
Relationship between particle size and *D*. **(A)** Zp of the NPs was kept the same. **(B)** Surface charge of the NPs was kept the same.

The relationship between *D* and the diameter of charged nanoparticles is plotted in Fig. 6 (A). The results show that with the same Zp, *D* is positively correlated to the particle diameter when the particle diameter is less than 90 nm. However, it is understood and also has been experimentally demonstrated that normally the smaller the particle is, the higher *D* it should possess [67]. Nevertheless, it should be noted that although Zp, or the effective surface charge density (*δ*_eff_) according to Eq. (8), is identical, the total surface charge is varying when the diameter changes. The increase of electrostatic forces may be, therefore, the reason why the *D* increases with the increase of diameter when Zp is kept the same.

To test this hypothesis, another parametric study was performed. In this group, diameters of the NPs are still 20 nm, 40 nm, 60 nm, 80 nm, 90 nm, and 98 nm, but the surface charge, rather than Zp, of all the NPs was kept the same, which was -200 *e*, where *e* is the elementary charge. The results are shown in Fig. 6 (B). The calculated *D* under this condition is negatively correlated to the particle diameter, which is then in accord with the above analysis.

It is also worth mentioning the existence of the size threshold (90 nm in this model), which indicates further increasing of particle size will decrease the *D* if the particle size has been very close to the width of ECS; even so, NPs as large as 98 nm still present considerable *D* in the WM and can easily pass through the gaps and if they are negatively charged.

Therefore, given the surface charge state of NPs is technically characterized by Zp, it could be concluded that, for identical negatively charged NPs, particle size is also positively correlated with their *D*s before reaching a threshold that is determined by the microstructure. Moreover, this implies that when the particle size is smaller than this threshold, electrostatic forces dominate the diffusion behaviours of NPs; whereas if the particle size exceed the threshold, interactions between particles and the cell membranes could determine the fate of NPs.

### Impact of the microstructure

More precisely, the abovementioned behaviours of the NPs are also affected by the geometry of brain tissues. Therefore, simulations are conducted to understand the impact of brain microstructure on particle size-*D* and Zp-*D* relationships.

**Fig. 7:**
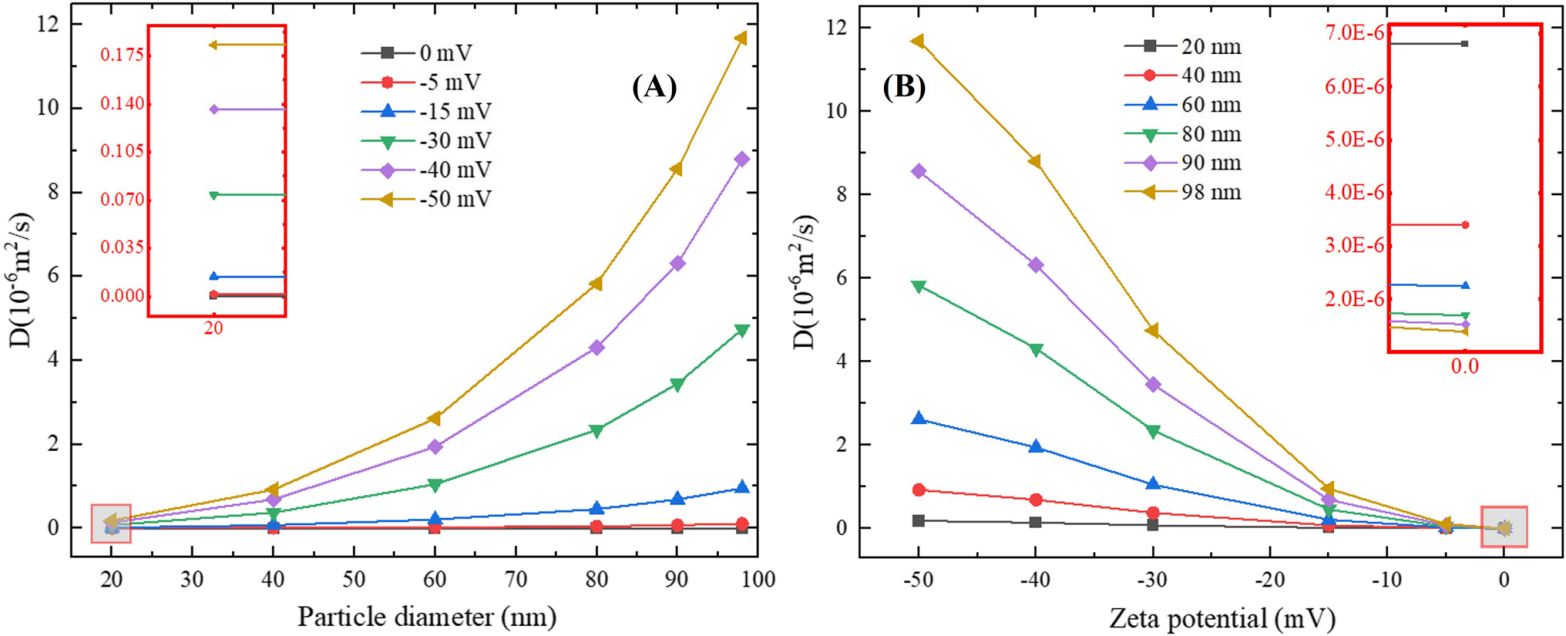
Diffusion of NPs in IF. **(A)** Relationship between particle size and *D*. **(B)** Relationship between Zp and *D*.

Diffusion of NPs with the same parameters in pure IF was simulated to investigate the difference between NP diffusion with and without axons. The results are shown in Fig. 7. Overall, the trends of particle size–*D* and Zp-*D* relations in pure IF are similar to that in WM. For negatively charged particles, both particle size and the absolute value of Zp correspond to an increase in *D*. In contrast to the NP diffusion in WM where a threshold of particle size presents, no such a threshold can be found for NPs diffusing in the pure IF. This finding demonstrates the importance of tissue microstructure in the NP transport.

**Fig. 8:**
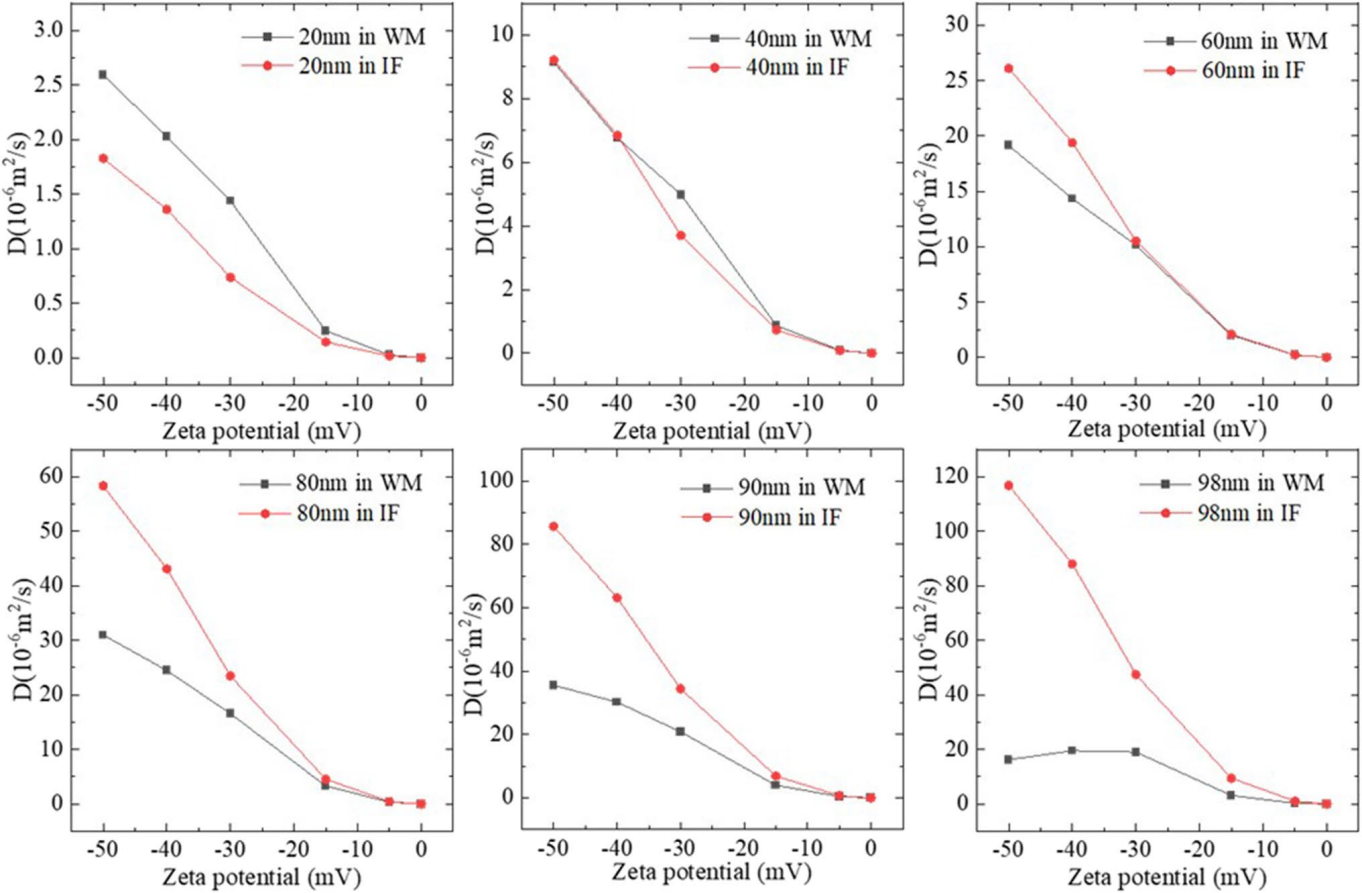
*D* of the NPs in WM and IF.

The values of *D* in WM and pure IF are also compared in Fig. 8. The results indicate that the impact of axons is more significant for the NPs with a larger size or higher negative charge. When the particle size is small or Zp is low, the difference between diffusion in brain and pure IF is limited, especially when the absolute value of Zp is smaller than 10 mV. This may imply that IF might be used as a replacement to measure the *D* of NPs in WM in the development of nano-drugs or nano-carriers if the NPs are uncharged or their Zp is low enough, i.e., between 0 mV and -10 mV.

There is also an interesting phenomenon that when particle size is smaller than 40 nm, the *D* in brain is even higher than the *D* in the pure IF while the intuitive understanding should be just the opposite. The particle-cell membrane interaction might be responsible for this. For large particles, especially those with a comparable dimension as the ECS width, repulsion/collision from the surrounding particles and cell membranes make it hard for the particles to pass through the narrow gaps. Therefore, *D*s of these NPs in the WM are certainly lower than *D*s in the pure IF. In contrast, the possibility for small NPs to hit the cell membrane is relatively low. Moreover, given both the cells and NPs are negatively charged, the force due to the electrostatic potential would accelerate the NP movement. As a result, the particles can present deeper penetration with higher *D*s.

## Discussion

If we only consider the effect of Zp on *D*, the results show that more negative Zp is better in general. However, studies also show that Zp is closely related to neuronal electrical activity [68]. Since aberrant neuronal electrical activity is associated with most neurological diseases [69, 70], it is crucial to also understand how Zp affects brain electrical function before determining the most appropriate Zp for the NPs. If one wants to increase *D* by charging the NPs and simultaneously avoid affecting neuronal electrical activity, low Zp might be an option, because the results in Fig. 5 show that higher Zp has limited effect on *D*. For example, when the absolute value of Zp raises from 0 to 5 mV, the *D* is increased by a factor of 10,000; after that, the values of *D* stay nearly in the same order of magnitude with more negative Zp. Results in Fig. 5 also show that even if the NPs were charged by just -5 mV, their *D*s could reach the level of 10^−8^m^2^/s, which is higher than the *D* of some plain anticancer drugs in IF, such as carmustine (10^−9^m^2^/s) and paclitaxel (10^−10^m^s^/s) [71, 72].

It is generally believed that smaller particles have more vigorous molecular thermal motion; thus their *D*s are higher. However, the results in our study show that for charged NPs with an identical Zp, the NPs’ effective diffusion coefficient can possibly be increased by enlarging the size. The reason is that the conventional understanding is based on electroneutral particles, whereas the nano-drugs and nano-carriers are usually charged for pharmacological applications. With the same Zp, larger particles have more surface charge, so the Coulomb force between particles is bigger, and thus accelerating the NP movement. It is worth explaining that because the surface charge of NPs can only be characterized by Zp in practice, Zp was set as an influential factor to do parametric study in this work to potentially provide practical suggestions. In fact, Zp that reflects the surface charged density is not an independent parameter. As shown in Eq. (8), the surface charged density is a function of both the particle size and surface charge. The finding on the size-*D* relationship indicates that particle interactions caused by Coulomb force overwhelms the thermal motion led by Brownian force for the diffusion of charged particles. This, in fact, provides a new insight into promoting *D* while limiting the effect of Zp on brain electrical function, which is to increase the size of the particle. However, the proper size of NPs cannot be determined simply by its impact on *D*, as particle size is also one of the key parameters for cell-uptake [7]. On the other hand, as all experimental studies done to date for measuring nanoparticle diffusion in the brain have been performed with very low concentrations of nanoparticle solutions [13–16], whether to consider particle-particle interaction in the corresponding mathematical model might be controversial. The results in this study show that even in very low particle concentration solution, particle-particle electrostatic interaction can still dominate the diffusion of NPs.

It is also important to know the maximum size of particles that can diffuse in the WM. Results in this study show that NPs as large as the width of ECS can transport in WM with a considerable *D* if the particle is negatively charged. For example, while the average ECS width in the present model is 100 nm, the *D* of 98 nm particle with Zp of -5 mV can reach 2.59 × 10^−8^ m^2^/s. Of course, if the highest *D* is needed, it is suggested that the particle diameter should be around 90 nm, as shown in Fig. 5. However, as stated in section 2.2, due to the structural difference of brain tissue caused by region, age, gender, and health condition, more specific data is needed to update the geometric model before giving patient-specific suggestions.

It is worth mentioning that while the length-scales in this study is similar to that in some experiments [14, 15], both of which are in the μm range; the time-scales considered in this study (of order of ms) is lower than those probed by the experiments (of order of s). In reality, the time-scales depend on the size of the area monitored as a representative volume and the diffusion velocity of the NPs, so the specific monitoring time in this study were different for different NPs. Of course, the monitoring time should be long enough to let the NPs reach a steady diffusion status. In our study, due to the finite diffusion area in the RVE (18 μm×18 μm), the monitoring time was constrained by the requirement to ensure all the monitored NPs were diffusing inside the RVE without being affected by the boundaries. It was found that the NPs’ diffusion could achieve stability at the scale of ms in our RVE (shown as stage II, Fig. 3 in the manuscript). Therefore, we chose the data on this stage to derive the effective diffusion coefficients. Time scale is also a vital index in determining the terms of the mathematical equations. The short time-scales targeted in the present study (ms), in fact, is also a reason why some of the factors in real situation were not considered in this study (see section 2.1.5), because it may be too short for the change of local gradients to develop. This should lead to the difference between short-term diffusivity and long-term diffusivity. These gradients induced by cell activities may need to be considered when tackling problems at longer time-scales. Our future work will also aim to model both short-term and long-term diffusivity.

Although some details of the real brain microstructure have been omitted in both mathematical and geometrical models, the newly established modelling system can reproduce experimental results, as shown in Table II. This means that the present modelling method could be used to predict *D* for nano-carriers or nano-drugs in brain tissue. If combined with mixture theory to also consider NPs transport in the surrounding capillary and across the vessel wall [73, 74], this method could further provide a more systematic prediction for nano-drug delivery in the brain.

Finally, some hypotheses in the models need further discussions. (i) Although statistical data was used to reconstruct the stochastic model, the microstructure of WM is still idealized. Components in the ECM which may affect the NPs transport, and geometrical anisotropy that may redirect the NPs movement are not considered in the present geometric model. The distribution of the axons is also more disordered in the real brain. Therefore, the present study is more suitable to provide qualitative information and perform comparative studies; furthermore, the prediction accuracy would be further improved by exploring more realistic geometric models [75, 76]. (ii) Limited by the geometry, the NPs in this study are assumed to be spherical, so their size is determined by the diameter. However, other functional NPs have been developed with different shapes, such as nanotube, nanodisk, naoneedle, plateloid, and ellipsoid [77]. Shape, especially aspect ratio, is also a key parameter in determining *D* because it can not only change the hydraulic diameter but also influence charge distribution, charge density and apparent surface charge of NPs. For example, Cognet and co-workers have shown single-wall carbon nanotubes (more than 100 nm of length and 1 nm in diameter) can readily diffuse in brain slices [64]. Effects of particle size and Zp on *Ds* of these types of NPs may be different from those evaluated in the present study. However, the proposed mathematical models could be readily adapted to investigate the transport of NPs with other shapes in the brain. (iii) The cell-uptake of NPs was not considered in this model. The charge states of NPs could help to tune the interaction between the NPs and the cells. For example, Tatur et al. [78] found that positively charged NPs can easily pass through the membrane, embedding themselves within the lipid bilayer and destabilising the entire membrane structure. If the concentration is high, these NPs could kill the cell by destroying the cell membrane. In contrast, negatively charged NPs nearly do not penetrate the lipid membrane so they are suitable to serve as drug carriers [78]. Lower-scale simulation tools, such as coarse-grained molecular dynamics (CGMD) simulations, are capable of modelling these more detailed particle-cell interactions [79]. Thus, combing the present method with CGMD will further empower this framework and provide more precise suggestions for the drug development and pharmaceutical research.

## Conclusions

In this study, a modelling framework is established to simulate the NP movement in brain WM microstructure. It is first validated by comparing with experimental data, and then applied to investigate the effects of NP size and surface charge on the NP diffusion coefficient. Results show that Zp has a positive effect on the *D*s of negatively charged NPs in WM until reaching a size threshold which depends on the WM microstructure. It is important to note that increasing the NP size is possible to simultaneously raise the total surface charge, and thereby increases the *D* of the charged particle. This provides an important step to understand how particle size and Zp affect the *D* of NPs in WM, which could help to improve the design efficiency of nano-drugs and nano-carriers to treat brain diseases. Furthermore, the developed geometric and mathematical model provides a new technique that can be potentially used to predict drug delivery in the brain and other tissues with similar (fibrous and anisotropic) microstructure.

## Abbreviations

BBB: Brain-blood barrier
CSF: Cerebrospinal fluid
*D*: Effective diffusion coefficient
ECS: Extracellular space
ECM: Extracellular matrix
GM: Grey matter
IF: Interstitial fluid
MSD: Mean square displacement
NP: Nanoparticle
WM: White matter
Zp: Zeta potential
